# Tailoring the Morphology of Monodisperse Mesoporous Silica Particles Using Different Alkoxysilanes as Silica Precursors

**DOI:** 10.3390/ijms241411729

**Published:** 2023-07-21

**Authors:** Fabio Fait, Stefanie Wagner, Julia C. Steinbach, Andreas Kandelbauer, Hermann A. Mayer

**Affiliations:** 1 Institute of Inorganic Chemistry, University of Tübingen, Auf der Morgenstelle 18, 72076 Tübingen, Germany; fabio.fait@reutlingen-university.de (F.F.); stefaniewagnersb@googlemail.com (S.W.); julia.steinbach@reutlingen-university.de (J.C.S.); 2 Process Analysis and Technology (PA&T), Reutlingen Research Institute, Reutlingen University, Alteburgstrasse 150, 72762 Reutlingen, Germany; andreas.kandelbauer@reutlingen-university.de; 3 Institute of Wood Technology and Renewable Materials, Department of Material Sciences and Process Engineering (MAP), University of Natural Resources and Life Sciences, Gregor-Mendel-Strasse 33, 1180 Vienna, Austria

**Keywords:** mesoporous silica microspheres (MPSMs), hard template method, high-performance liquid chromatography (HPLC)

## Abstract

The hard template method for the preparation of monodisperse mesoporous silica microspheres (MPSMs) has been established in recent years. In this process, in situ-generated silica nanoparticles (SNPs) enter the porous organic template and control the size and pore parameters of the final MPSMs. Here, the sizes of the deposited SNPs are determined by the hydrolysis and condensation rates of different alkoxysilanes in a base catalyzed sol–gel process. Thus, tetramethyl orthosilicate (TMOS), tetraethyl orthosilicate (TEOS), tetrapropyl orthosilicate (TPOS) and tetrabutyl orthosilicate (TBOS) were sol–gel processed in the presence of amino-functionalized *poly* (glycidyl methacrylate-*co*-ethylene glycol dimethacrylate) (*p*(GMA-*co*-EDMA)) templates. The size of the final MPSMs covers a broad range of 0.5–7.3 µm and a median pore size distribution from 4.0 to 24.9 nm. Moreover, the specific surface area can be adjusted between 271 and 637 m^2^ g^−1^. Also, the properties and morphology of the MPSMs differ according to the SNPs. Furthermore, the combination of different alkoxysilanes allows the individual design of the morphology and pore parameters of the silica particles. Selected MPSMs were packed into columns and successfully applied as stationary phases in high-performance liquid chromatography (HPLC) in the separation of various water-soluble vitamins.

## 1. Introduction

The introduction of high-performance liquid chromatography (HPLC) has enabled a rapid chemical analysis and separation process for substances and end products. The wide range of applications extends from small molecules [1,2,3] to pharmaceuticals [4,5], food and environmental analysis [6,7,8], long-chain polymers [9,10] and biomolecules [11,12,13]. The most common material in HPLC columns are spherical silica particles because of their mechanical robustness. Moreover, silica particles possess reactive groups on their surface, by which a variety of functionalizations allow a fine tuning of the particle features [14,15,16]. Characteristics such as particle size, dispersity, pore structure and surface functionalization influence their chromatographic properties such as selectivity, analysis time, plate number and back pressure. Due to their high specific surface area, fully porous silica particles in the µm range have proven successful in HPLC [17,18,19].

Probably the best-known representation of spherical silica networks is the silica material obtained from the Stoeber process [20]. Non-porous spherical silica particles in the range of 10 to 500 nm can be formed by ammonia-catalyzed hydrolysis and the condensation of molecular alkoxysilanes. With semi-batch processes and the addition of electrolytes, the particle size can be increased into the µm range [21,22,23,24].

The hydrolysis rates of the alkoxysilanes are crucial for the size of the siloxane network. The hydrolysis rates are controlled by different parameters like the temperature; the chain length and branching of the alkoxysilane; NH_3_ and H_2_O concentrations; and the chain length and branching of the alcohol, which is used as a solvent [20,25,26,27,28,29]. Under basic conditions, an increase in the chain length of the alkyl groups of the alkoxysilane leads to a decrease in the rate of hydrolysis. The increasing steric hindrance and inductive effects of the alkyl groups increase the electron density at the silicon atom and make a nucleophilic attack more difficult. In addition, the decreasing polarity of the alkoxysilanes can lead to phase separation, depending on the solvent applied [29,30,31,32]. The NH_3_ and H_2_O concentrations influence the equilibrium reactions of hydrolysis and condensation. While under basic conditions the condensation of hydrolyzed alkoxysilanes is extremely fast, higher concentrations of H_2_O affect the hydrolysis positively but the condensation negatively [28,29,31,33,34]. Overall, the Stoeber process provides non-porous silica nanoparticles with narrow size distributions. However, the preparation of monodisperse silica particles in the micrometer range is challenging, and the obtained particles remain nonporous.

The preparation of mesoporous silica microspheres (MPSMs) with narrow size distributions remains challenging [35,36,37,38,39,40]. Recently, for the synthesis of MPSMs, a promising protocol has been developed that employs functionalized porous *poly* (glycidyl methacrylate-*co*-ethylene glycol dimethacrylate) polymer particles (*p*(GMA-*co*-EDMA)) as hard templates in the presence of the basic hydrolysis and condensation of TEOS [35]. *p*(GMA-*co*-EDMA) functionalized with trimethylamine, (3-amino propyl) triethoxysilane or tetra ethylene pentamine (TEPA) provides excellent environments to deposit silica nanoparticles (SNPs) in the pores of the template, where they form a silica network [36,37,38]. The best matches of the templates are achieved if the rate of the growth of the SNPs and their rate of diffusion into the template pores are well balanced. Moreover, the particle and pore properties correlate with the size of the SNPs, which depends on the sol–gel conditions (see above) and the template [39,40].

**Scheme 1:**
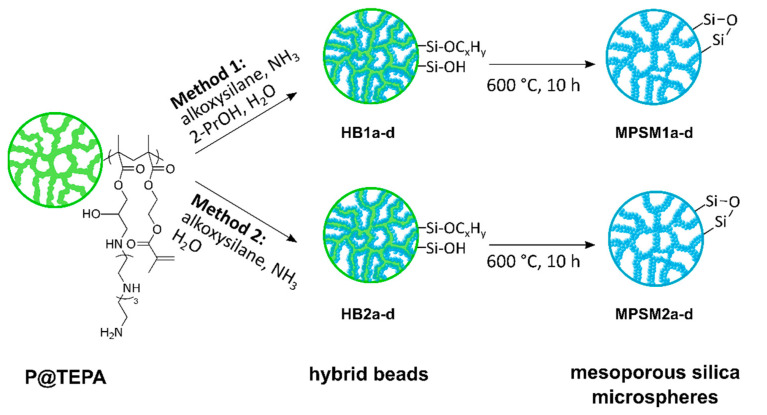
Synthesis of mesoporous silica microspheres **MPSM1a-d** and **MPSM2a-d** (TMOS = **a**, TEOS = **b**, TPOS = **c** and TBOS = **d**)**.**

In this study, we focus on the sol–gel conditions during the formation of MPSMs via the hard template method. Therefore, we investigated the influence of alkoxysilane precursors, which differ in their rates of hydrolysis on the particle and pore properties of MPSMs. For this, the sol–gel process of the precursors is carried out under basic conditions in the presence of the tetraethylenepentaamine-functionalized *p*(GMA-*co*-EDMA) template P@TEPA (Scheme 1). The size of the silica nanoparticles that accumulate in and on the template depends on the hydrolysis rate of the precursors. The *p*(GMA-*co*-EDMA)/SiO_2_ hybrid beads (HB) and the MPSMs are characterized for their particles and pore properties by scanning electron microscopy, thermogravimetric analysis, and nitrogen adsorption/desorption measurements. Finally, selected MPSMs are functionalized with trimethoxy (octadecyl) silane and applied as stationary phases in the separation of different water-soluble vitamins.

## 2. Results and Discussion

### 2.1. Preparation and Characterization of MPSM1a-d and MPSM2a-d

Monodisperse tetraethylenepentamine (TEPA)-functionalized *p*(GMA-*co*-EDMA) served as the template in the preparation of all MPSMs discussed here. The characteristic features of this P@TEPA template are a diameter of 6.0 ± 0.5 µm, a median pore diameter of 14.4 nm and a pore volume of 0.24 mL g^−1^ (Figures S2 and S3, Supporting Information).

In the presence of the P@TEPA template, a basic sol–gel process was performed with the four different alkoxysilanes, TMOS (**a**), TEOS (**b**), TPOS (**c**) and TBOS (**d**), in 2-propanol and H_2_O as solvents (Methods 1 and 2, Scheme 1). In Method 1, all reactants except the template are soluble in 2-propanol. Under these conditions silica nanoparticles (SNP) are formed in the continuous phase, which diffuse into the template and accumulate in the pores and on the surface [37,38,39,40]. The size of the SNPs depends on the hydrolysis and condensation rates of the precursors. With the fastest rate of hydrolysis, TMOS forms the largest SNPs, which accumulate in the pores (Figure S4, **HB1a**). Moreover, the hydrolysis and condensation rates of TMOS are so high that some of the SNPs become too large (~60 nm) to enter the template network. These secondary particles remain in the continuous phase and are mostly removed from the reaction mixture during the purification process, while some of them are left at the template surface (Figure S4, **HB1a**). With TEOS as the precursor, particle formation is already four times slower than for TMOS [32], resulting in smaller SNPs that easily penetrate the porous network of the template and form **HB1b** (Figure S4). The longer alkoxy chains of the TPOS and TBOS alkoxides decrease their hydrolysis rates further. Thus, less silica species are available for condensation to build SNPs. The accumulation of SNPs in the template is now difficult, and SNPs are hardly observed on the surface of the hybrid particles (Figure S4, **HB1c** and **HB1d**). Overall, the size of the SNPs decreases with the rates of hydrolysis in the TMOS > TEOS > TPOS > TBOS series (Figure S4), and the incorporation of silica into the pores of the template is best achieved for TEOS.

In Method 2, the sol–gel process is carried out in H_2_O, which reduces the differences of the kinetic effects of the hydrolysis and condensation of the four different alkoxysilanes (Method 2, Scheme 1). This should have an impact on the SNP formation and the incorporation of silica into the pores of the template. Ammonia as catalyst is added after 24 h of stirring to enable the nonpolar precursors to diffuse into the template network. Condensation does not start until NH_3_ is added. After another 24 h, the hybrid particles **HB2a-d** are obtained (Figure S5 and Table 1). As a consequence of the reduced rate of the hydrolysis of TMOS, no secondary particles are observed. The particles **HB2a** grow by 0.7 µm and **HB2b** by 0.3 µm and are thus larger than **HB1a-d**. In contrast to particles prepared by Method 1, a more edgy morphology of the hybrid materials is achieved. For TPOS (**HB2c**) and TBOS (**HB2d**) as precursors, there are no changes in size and morphology compared to the template. 

**Figure 1:**
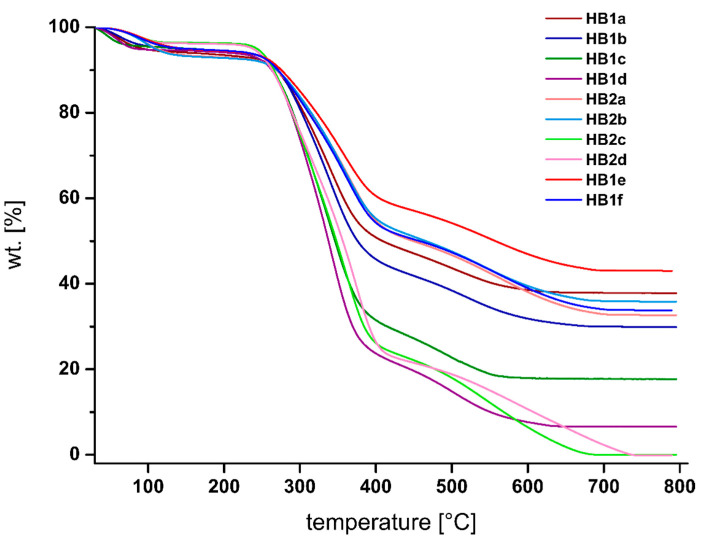
TGA measurements of hybrid beads **HB1a-f** and **HB2a-d**.

The thermal degradation behavior of the hybrid beads **HB1a-d** and **HB2a-d** compares well with that reported earlier (Figure 1) [41]. After the loss of surface water, the degradation processes of the polymer backbone led to a complete decomposition of the template and allowed the determination of the silica content of the hybrid beats. Here the hybrid particles **HB1a** contain the highest quantity of silica (37.8 %). The amounts of silica of **HB1b** (29.9%), **HB1c** (17.7%) and **HB1d** (6.6%) correlate with their decreasing hydrolysis rates. The amounts of SiO_2_ in **HB2a** and **HB2b** (32.7% and 35.8%, respectively) differ little (Figure 1). Due to the suppressed hydrolysis in H_2_O, the hydrolysis rates of TMOS and TEOS are comparable. Thus, similar amounts of SiO_2_ are deposited. The percentage of incorporated silica in the hybrid particles correlates well with the particle size of the resulting MPSMs (Table 1). Thermogravimetric analyses of **HB2c** and **HB2d** result in only very small amounts of SiO_2_. This is traced back to the poor miscibility of the alkoxysilanes TPOS and TBOS with water. Thus, only small amounts of SNPs are generated during the reaction.

**Figure 2:**
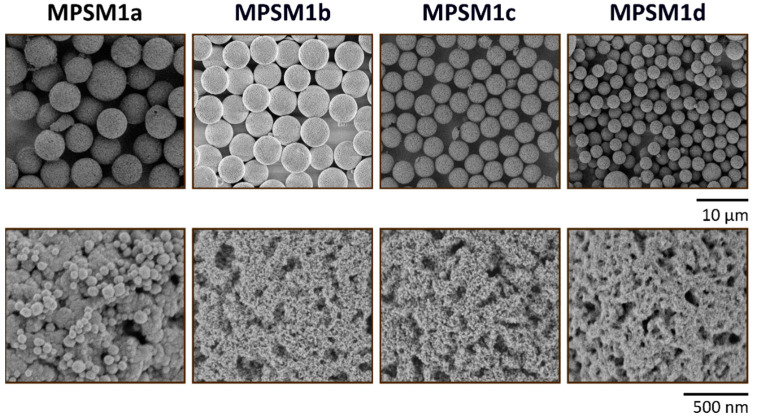
SEM images of mesoporous silica microspheres **MPSM1a-d** with 2000× magnification (**top row**) and 50,000× magnification (**bottom row**).

**Figure 3:**
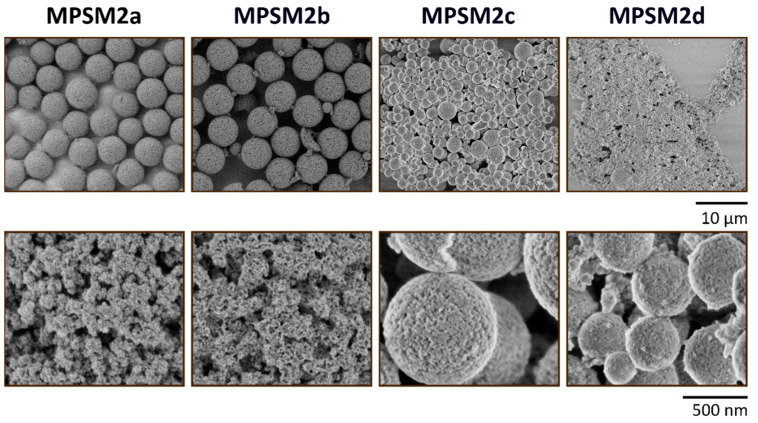
SEM images of mesoporous silica microspheres **MPSM2a-d** with 2000× magnification (**top row**) and 50,000× magnification (**bottom row**).

The calcination of the hybrid beads **HB1a-d** and **HB2a-d** for 10 h at 600 °C removed the organic polymer template and released the monodisperse mesoporous silica microspheres **MPSM1a-d** (Figure 2) and **MPSM2a-d** (Figure 3). The nanoparticulate morphology of the MPSMs is comparable to that of their corresponding hybrid beads. The particle size of the MPSMs decreases with the decreasing hydrolysis rate of the precursors. Thus, while **MPSM1a** (6.0 µm) and **MPSM1b** (5.5 µm) represent the size of the template quite well, the sizes of **MPSM1c** (3.6 µm) and **MPSM1d** (2.2 µm) are strongly reduced. Consequently, only TMOS and TEOS map the template to 100% and 92%, respectively, while, for TPOS and TBOS, the template is mapped to only 60% and 37%, respectively. The particle sizes of **MPSM2a** and **MPSM2b** are 5.9 µm and 6.0 µm, respectively, and completely replicate the template. For **MPSM2c** and **MPSM2d**, 800 nm and 500 nm polydisperse porous silica particles are generated.

**Figure 4:**
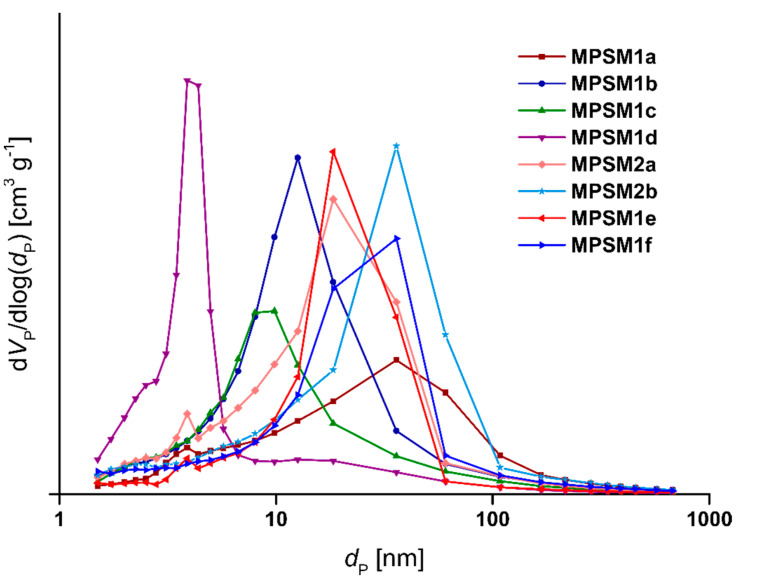
Pore size distributions of MPSM1a-f and MPSM2a-b.

The pore properties of the MPSMs were determined via nitrogen adsorption/desorption measurements and are listed in Table 1. The corresponding pore size distributions are shown in Figure 4. Here, the median pore size of the MPSMs decreases and the specific surface area increases with the decreasing hydrolysis rates of the precursors. This result is consistent with the size of the SNPs that form the silica network. Large SNPs generate large pores of the MPSMs, while small SNPs result in smaller pores [38,40]. Therefore, the median pore size becomes smaller in the order of **MPSM1a** (23.6 nm), **MPSM1b** (11.3 nm), **MPSM1c** (8.8 nm), and **MPSM1d** (4.0 nm). As smaller pores form larger specific surface areas, the highest specific surface area is obtained for **MPSM1d**, and the lowest specific surface area is obtained for **MPSM1a**. The sol–gel process according to Method 2 leads to an edgier morphology for **MPSM2a** and **MPSM2b**, resulting in larger surface areas compared to **MPSM1a** and **MPSM1b**. The pore volume of the MPSMs differs between 0.5 mL g^−1^ and 0.9 mL g^−1^.

### 2.2. Preparation and Characterization of MPSM1e and MPSM1f

**Figure 5:**
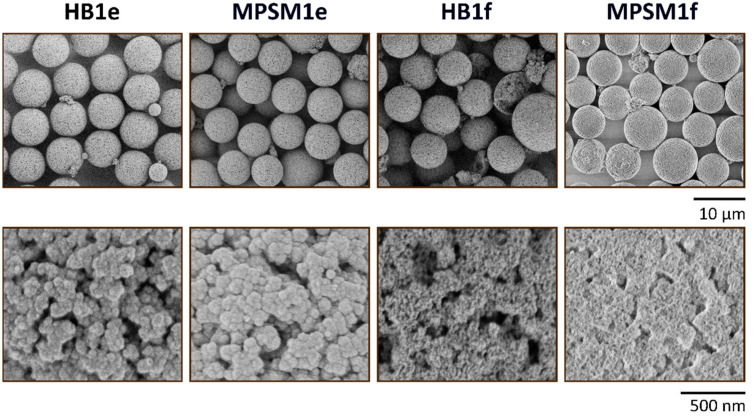
SEM images of hybrid beads **HB1e** and **HB1f** and corresponding **MPSM1e** and **MPSM1f** with 2000× magnification (**top row**) and 50,000× magnification (**bottom row**).

The properties of the MPSMs are controlled by the hydrolysis rate of the precursors and the solvent medium. TMOS produces non-porous secondary particles while TPOS does not fully map the size of the template if the sol–gel process is carried out in 2-propanol and H_2_O. To avoid this unwanted behavior, the two precursor combinations of TMOS with TEOS (**MPSM1e**) and TPOS with TEOS (**MPSM1f**) were applied in a sol–gel process in the presence of a P@TEPA template with a diameter of 7.2 µm. The new HBs and MPSMs are shown in Figure 5. Interestingly, no secondary particles are observed for **HB1e** and **MPSM1e**. The **HB1e** particles have the highest silica content of all hybrid particles, and the corresponding silica microspheres have a nanoparticulate surface and exhibit a size of 7.3 µm (Table 1). Thus, they completely map the template without the negative effects of the high hydrolysis rate of TMOS. With a median pore size of 16.6 nm, this is in between that of **MPSM1a** and **MPSM1b**. This results in SNPs in the continuous phase that are smaller than those of **MPSM1a** and larger than those of **MPSM1b**. The combination of TEOS and TPOS leads to the particles **HB1f** and **MPSM1f**. The resulting silica materials have a size of 6.6 µm, representing 92% of the template. Interestingly, the median pore size of 15.6 nm and the pore volume of 1.06 mL g^−1^ are larger than the pore properties of **MPSM1b**, for which only TEOS was used. Compared with **MPSM1c**, the template is better replicated in **MPSM1f**.

### 2.3. Chromatographic Measurements of MPSM1b

For the use of MPSMs as a stationary phase in high-performance liquid chromatography, high monodispersity is required to achieve efficient separation. **MPSM1b** particles were chosen based on their particle size and monodispersity to investigate their suitability as a stationary phase in HPLC. Therefore, **MPSM1b** particles were functionalized with trimethoxy (octadecyl) silane and packed in a 250 mm × 4.6 mm stainless steel column with acetone as the slurry and methanol/water (85 v.%/15 v.%) as the pressure medium. 

**Figure 6:**
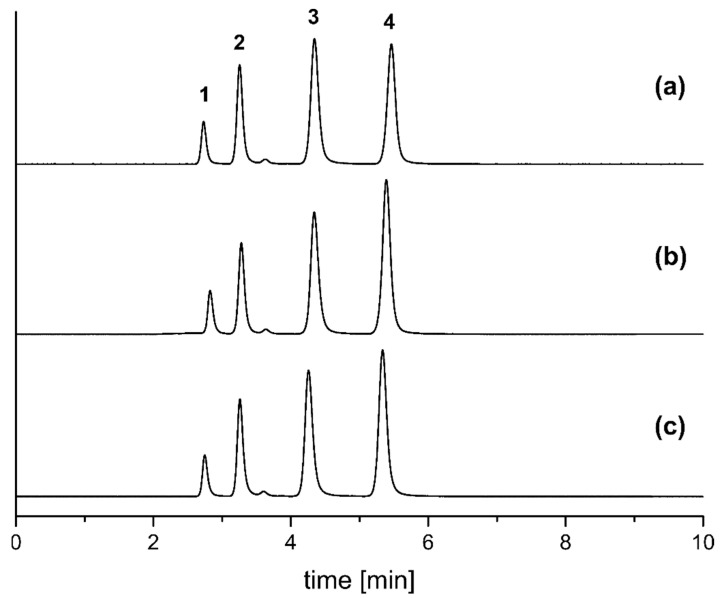
Reproducibility of chromatographic properties from three different batches (**a**–**c**) of **MPSM1b-C_18_** synthesized according to Method 1. Chromatographic settings: column dimension: 250 × 4.6 mm; mobile phase: MeOH/H_2_O (85 v.%/15 v.%); analytes: uracil (**1**), phenol (**2**), *N*,*N*-diethyl-*m*-toluamide (**3**) and toluene (**4**); flow: 1 mL min^−1^; injection volume: 5 µL; UV detection: 254 nm.

The reproducibility of the synthesis of **MPSM1b** in its chromatographic properties is shown in Figure 6. The particles of three different batches with the same reaction conditions were packed in 250 mm × 4.6 mm stainless steel columns and examined for their chromatographic properties. As can be seen in Figure 6, the particles of all three batches show the same retention behavior of the test mixture. Moreover, even after one hundred injections, the retention times of toluene and uracil did not change (Supporting Information Table S1). This indicates the good stability of the stationary phase **MPSM1b-C_18_**.

**Figure 7:**
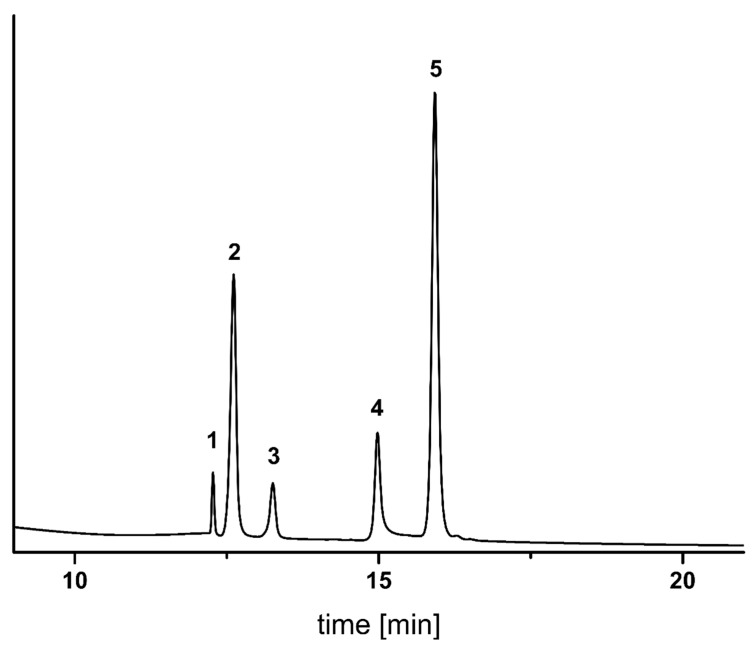
Separation of five water-soluble vitamins B_1_ (**1**), B_3_ (**2**), B_5_ (**3**), B_9_ (**4**) and B_12_ (**5**). Chromatographic settings: column dimension: 250 × 4.6 mm; eluent A: H_2_O with 0.025 v.% TFA; eluent phase B: ACN; gradient (A/B): 5 min (100/0), 11 min (75/25), 19 min (60/40), 20 min (60/40), 21 min (100/0) and 25 min (100/0); flow: 0.8 mL · min^−1^; injection volume: 20 µL; column temperature: 30 °C; UV detection: 210 nm.

The successful separation of five water-soluble vitamins is shown in Figure 7. A gradient from eluent A, consisting of water containing 0.025% TFA, to eluent B, consisting of acetonitrile (ACN), was used for the separation. An initial isocratic step for five minutes with eluent A is followed by an increase from eluent B to eluent A to 25/75 (v.%/v.%) in six minutes, as proposed by Heudi et al. [42]. This is followed by a second gradient on eluent B to eluent A 40/60 (v.%/v.%) in eight minutes, holding this for an additional minute. Then, the initial conditions are restored in one minute and equilibrated for four minutes. The vitamins were baseline separated and assigned based on single measurements of the analytes. The elution order is vitamin B_1_ < B_3_ < B_5_ < B_9_ < B_12_ as detected at 210 nm.

## 3. Materials and Methods

### 3.1. Chemicals

Tetraethyl orthosilicate (TEOS), tetramethyl orthosilicate (TMOS) and trimethoxy (octadecyl) silane (ODTMS) were obtained from abcr GmbH (Karlsruhe, Germany). Ammonia (28–30% aqueous solution), tetrapropyl orthosilicate (TPOS) and tetrabutyl orthosilicate (TBOS) were purchased from Alfa Aesar (Schwerte, Germany). Ethanol, hydrochloric acid, 2-propanol, triethylamine and the water-soluble vitamins (B_1_, B_3_, B_5_, B_9_ and B_12_) were bought from Sigma-Aldrich (Taufkirchen, Germany). Acetonitrile (ACN), trifluoro acetic acid (TFA) and water (all HPLC grade) were purchased from Fisher Scientific (Schwerte, Germany). Toluene and deionized water were cleaned using a solvent purification system. The test mixture (uracil, phenol, *N*,*N*-diethyl-*m*-toluamide and toluene) for column characterization was provided by Dr. Maisch HPLC, (Ammerbuch, Germany).

### 3.2. Characterization

For the evaluation of the morphology, particle size and dispersity, SEM images were acquired using a Hitachi SU8030 (Krefeld, Germany). The mean particle diameter was obtained by calculating at least 400 particles from SEM images and is expressed in µm. The pore parameters of the materials are determined by nitrogen adsorption on a BELSORP MiniX from Microtrac Retsch GmbH (Haan, Germany). The sample preparation was carried out on a BELSORP VACII (Microtrac Retsch GmbH, Haan, Germany). For that, the silica materials were heated for 3 h at 300 °C, and a vacuum of 2 × 10^−2^ mbar was used to remove possible physisorbed residues and to achieve a reproducible equilibrium [43]. Adsorption and desorption isotherms were performed at 77 K. For the determination of the specific surface area, the adsorption isotherms were evaluated by the Brunauer–Emmet–Teller (BET) method, and for the pore volume (single point measurement at p/p_0_ = 0.95) and pore size distributions, the desorption isotherms were evaluated by the Barrett–Joyner–Halenda (BJH) method using BELMaster 7 software [44,45]. The amount of SiO_2_ was determined after thermogravimetric measurements on a Mettler Toledo TGA/DSC. Samples were weighed in an aluminum vessel and measured at a heating rate of 5 K min^−1^ and synthetic air (50 mL min^−1^).

Analytical high-performance liquid chromatography of water-soluble vitamins was performed on an Agilent 1100 series system from Agilent Technologies (Waldbronn, Germany), which consisted of a quaternary pump with degasser, an autosampling system, a column oven and a diode array detector. Instrument control, data acquisition and automated data analysis was performed by the OpenLAB CDS (Rev. C.01.07 SR3 software, Agilent Technologies, Walbronn, Germany). A running gradient of eluent A consisting of water and 0.025 v.% TFA to eluent B consisting of acetonitrile was used according to Heudi et al. [42] The vitamins B_1_, B_5_ and B_12_ (1 mg mL^−1^), B_3_ (0.5 mg mL^−1^) and B_9_ (2 mg mL^−1^) were dissolved in water.

### 3.3. Syntheses

Monodisperse porous *p*(GMA-*co*-EDMA) particles were prepared by a seed suspension polymerization of glycidyl methacrylate and ethylene glycol dimethacrylate in the presence of monodisperse polystyrene particles (1.5 ± 0.1 µm, Figure S1, Supporting Information) [35,36,46]. Then, the *p*(GMA-*co*-EDMA) particles were functionalized with TEPA according to previous reports (for details, see Supporting Information, Figures S1–S3 [36,38,39]) to generate P@TEPA template particles. A nitrogen content of 2.4% and spectroscopic analysis indicate successful functionalization.

#### 3.3.1. Preparation of Monodisperse Porous Hybrid Beads (HB1a-f and HB2a-d) and Mesoporous Silica Microspheres (MPSM1a-f and MPSM2a-d)

Method 1: An amount of 1 g of P@TEPA particles was dispersed in a mixture of 60 mL of 2-propanol and 7.5 mL of H_2_O. Then, 2.4 mL of TMOS (**a**), TEOS (**b**), TPOS (**c**) and TBOS (**d**) and 0.2 mL of an aqueous ammonia solution (28–30%) was added, and the mixture was stirred at 200 rpm for 24 h to produce hybrid beads **HB1a-d** (Table 1).

The hybrids **HB1e-f** were produced after 2.4 mL of TEOS and 1.5 mL of TMOS (**e**) or TPOS (**f**) and 0.2 mL of an aqueous ammonia solution (28–30%) were added to a dispersion of 1 g P@TEPA particles in 60 mL of 2-propanol and 7.5 mL of H_2_O. The mixture was stirred at 200 rpm for 24 h (Table 1).

Method 2: An amount of 1 g of P@TEPA particles was dispersed in 67.5 mL of H_2_O. Then, 2.4 mL of the corresponding alkoxysilane was added, and the mixture was stirred at 200 rpm. After 24 h, 0.2 mL of an aqueous ammonia solution (28–30%) was added, and the reaction was stirred for further 24 h at 200 rpm to produce hybrid beads **HB2a-d** (Table 1).

All hybrid beads were separated from their solutions, washed three times with EtOH and three times with H_2_O, and dried at 65 °C for 16 h. The resulting hybrid beads were calcinated at 600 °C for 10 h to provide the corresponding mesoporous silica microspheres **MPSMs** (Table 1).

#### 3.3.2. Octadecyl Functionalization of Mesoporous Silica Microspheres for Chromatographic Measurements

An amount of 5 g of silica particles **MPSM1b** was dispersed in 600 mL of hydrochloric acid (3.7%) and stirred for 3 h at 100 °C (200 rpm). The particles were separated from the solution, washed with EtOH and H_2_O until neutral and dried at 65 °C for 16 h. The particles were then dispersed in 75 mL of toluene; 25 mL of ODTMS and 0.5 mL of triethylamine were added; and the mixture was stirred at 100 °C (200 rpm) for 6 h. The particles were separated from the solution; washed three times with toluene, three times with EtOH and twice with MeOH; and dried at 65 °C for 16 h.

The functionalized particles were packed with acetone as slurry and MeOH/H_2_O (85 v.%/15 v.%) as pressure medium.

## 4. Conclusions

Monodisperse mesoporous silica microspheres (MPSM) can be tailored in their sizes and pore parameters via the hard template method. This is achieved if, at the stage of the hybrid bead syntheses, the sol–gel parameters are adjusted properly. This has been successfully demonstrated here by applying a basic sol–gel process with four different alkoxysilanes in the presence of functionalized *p*(GMA-*co*-EDMA) as the template. The SNPs grow at various rates and are thus incorporated into the template pores in non-uniform sizes, which is a consequence of the different hydrolysis and condensation rates of the alkoxysilane precursors. Thus, different amounts of silica are incorporated into the template, which has an impact on the final size of the MPSM. With TMOS and TEOS as precursors, the size of the template is reproduced, while TPOS and TBOS as precursors lead to much smaller MPSMs. Moreover, the various sizes of the incorporated SNPs generate different pore parameters. The larger the SNP, the larger the pores of the MPSM, which is important for HPLC applications. The silica particles synthesized with TEOS according to Method 1 were functionalized with trimethoxy (octadecyl) silane and used as the stationary phase in HPLC. The complete baseline separation of five water-soluble vitamins was achieved with these microspheres. The robustness of the synthesis of MPSMs in their chromatographic properties was demonstrated via HPLC using three different batches with a reversed phase test mixture.

## Figures and Tables

**Table 1:** Particle properties of hybrid beads and corresponding mesoporous silica microspheres.

	Particle Size	SiO_2_ Content		Particle Size	Median Pore Size	Pore Volume	Specific Surface Area
	(µm)	(%)		(µm)	(nm)	(mL g^−1^)	(m² g^−1^)
HB1a	6.3	37.8	MPSM1a	6.0	23.6	0.50	271
HB1b	6.3	29.9	MPSM1b	5.5	11.3	0.84	389
HB1c	6.3	17.7	MPSM1c	3.6	8.8	0.62	339
HB1d	6.3	6.6	MPSM1d	2.2	4.0	0.68	637
HB2a	6.7	32.7	MPSM2a	5.9	15.7	0.87	390
HB2b	6.2	35.8	MPSM2b	6.0	24.9	0.69	346
HB2c	6.0	0.01	MPSM2c	0.8	^1^	^1^	^1^
HB2d	6.0	0.01	MPSM2d	0.5	^1^	^1^	^1^
HB1e	7.1	43.0	MPSM1e	7.3	16.6	0.79	247
HB1f	8.6	33.8	MPSM1f	6.6	15.6	1.06	311

^1^ The poor yield did not allow us to record adsorption/desorption measurements.

## Data Availability

Data will be made available on request.

## Supplementary Materials

The following supporting information can be downloaded at https://www.mdpi.com/article/10.3390/ijms241411729/s1.
