# Supplementary material for: Tailoring the Morphology of Monodisperse Mesoporous Silica Particles Using Different Alkoxysilanes as Silica Precursors
Source: Int J Mol Sci. 2023 Jul 21;24(14):11729. doi: 10.3390/ijms241411729 (PMC10380632; doi:10.3390/ijms241411729)
Supplement: Supplementary file 1 [file ijms-24-11729-s001.zip › ijms-2476044-supplementary.pdf]

Modification of the morphology of monodisperse mesoporous silica particles using different alkoxysilanes.

Fabio Fait [1,2], Stefanie Wagner [1], Julia C. Steinbach [1,2], Andreas Kandelbauer [2,3] and Hermann A. Mayer [1,*]

1 Institute of Inorganic Chemistry, University of Tübingen, Auf der Morgenstelle 18, 72076 Tübingen, Germany; fabio.fait@reutlingen-university.de (F.F.); stefaniewagnersb@googlemail.com (S.W.); julia.steinbach@reutlingen-university.de (J.C.S.)

2 Process Analysis and Technology (PA&T), Reutlingen Research Institute, Reutlingen University,
Alteburgstrasse 150, 72762 Reutlingen, Germany; andreas.kandelbauer@reutlingen-university.de

3 Institute of Wood Technology and Renewable Materials, Department of Material Sciences and Process
Engineering (MAP), University of Natural Resources and Life Sciences, Gregor-Mendel-Strasse 33,
1180 Vienna, Austria

* Correspondence: hermann.mayer@uni-tuebingen.de

Chemicals

Polyvinyl alcohol (PVA, 87-89% hydrolyzed, mean average 88000 – 97000 g mol^{-1}), polyvinylpyrrolidone K_{30} (PVP, mean average 40000 g mol^{-1}) and K_{90} (mean average 360000 g mol^{-1}) from abcr GmbH. Ethyleneglycol dimethacrylate (EDMA) was bought from Acros Organics. Cyclohexanol and sodium dodecyl sulfate (SDS) were purchased from Carl Roth. Styrene was obtained by Fisher Chemicals. Dibenzoyl peroxide (BPO), dibutyl phthalate (DBP), glycidyl methacrylate (GMA) and tetraethylene pentamine (TEPA) were purchased from Sigma-Aldrich.

Synthesis of polystyrene

Monodisperse polystyrene particles with diameters of 1.5 ± 0.1 were prepared as reported earlier (**Figure S1**)

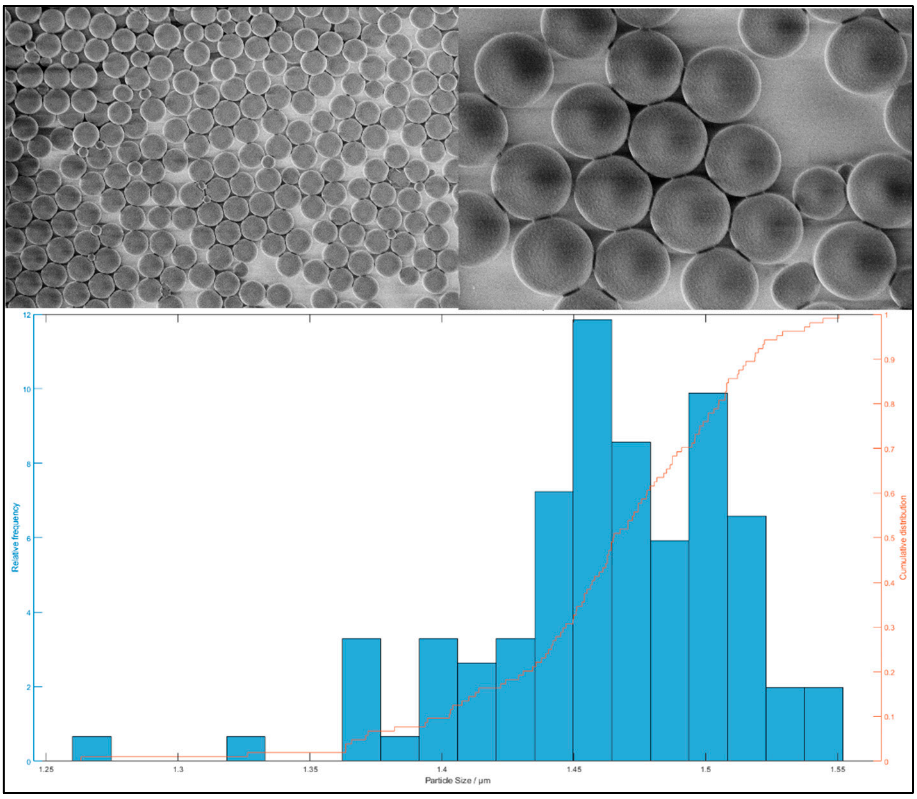

Figure S1. SEM images of applied polystyrene particles (top) and their particle size distribution (bottom).

Synthesis of P@TEPA

0.3 g of polystyrene particles and 5 mL of an aqueous SDS solution (0.25 wt.%) were dispersed in a 250 mL flask. 2 mL DBP were emulsified in 150 mL aqueous SDS solution (0.25 wt.%) with a homogenizer at 4500 rpm for 15 min and then added to the polystyrene suspension. The mixture was stirred for 24 h at 200 rpm. 6 mL GMA, 9 mL EDMA, 6 mL cyclohexanol, 16.5 mL toluene, 0.4 g BPO and 150 mL aqueous SDS solution (0.25 wt.%) were emulsified with a homogenizer at 4500 rpm for 15 min. This emulsion, a 150 mL aqueous PVA solution (2.3 wt.%) and the activated polystyrene particles were transferred to a 500 mL three-necked flask and stirred for 24 h at 200 rpm. Argon was passed into the reaction mixture for 30 minutes and then heated to 70 °C for 24 hours. The particles were separated from the solution, washed three times with EtOH and three times with H_2O and dried at 65 °C for 16 h. The average particle size of the *p*(GMA-*co*-EDMA) particles were 6.0 ± 0.5 µm (**Figure S2**).

5 g of *p*(GMA-*co*-EDMA) particles and 200 mL of H_2O were dispersed in a 500 mL flask. 7.5 of mL TEPA were added while stirring the suspension at 200 rpm. The reaction mixture was

heated to 80 °C for 24 h. The particles were separated from the solution, washed three times with EtOH and three times with H_2O and dried at 65 °C for 16 h.

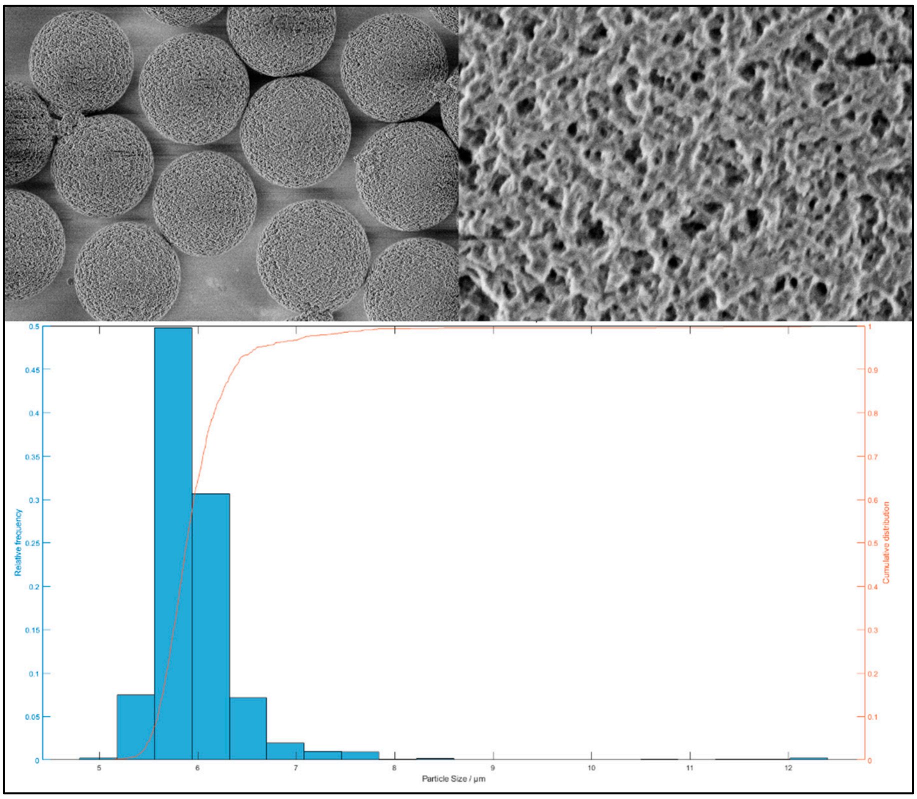

Figure S2. SEM images of the polymer templates *p*(GMA-*co*-EDMA) (top) and corresponding particle size distribution (bottom).

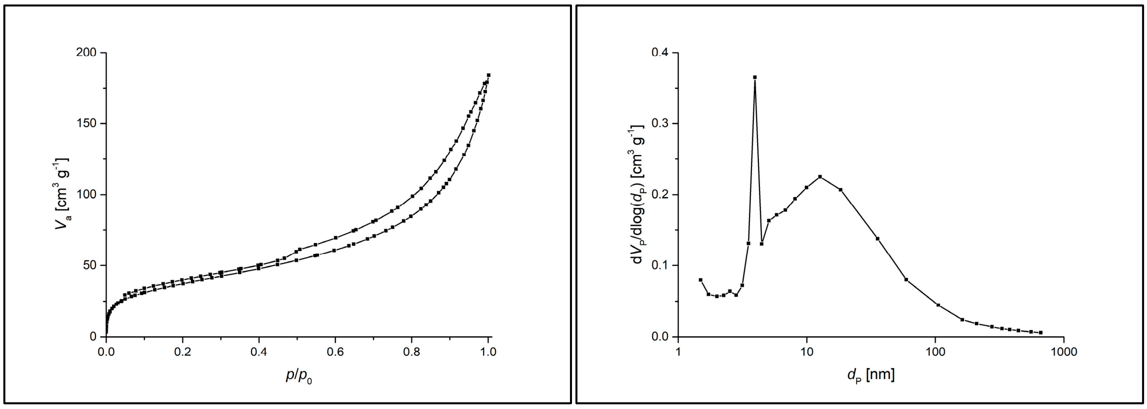

Figure S3. Isotherms (left) and pore size distribution (right) of *p*(GMA-*co*-EDMA).

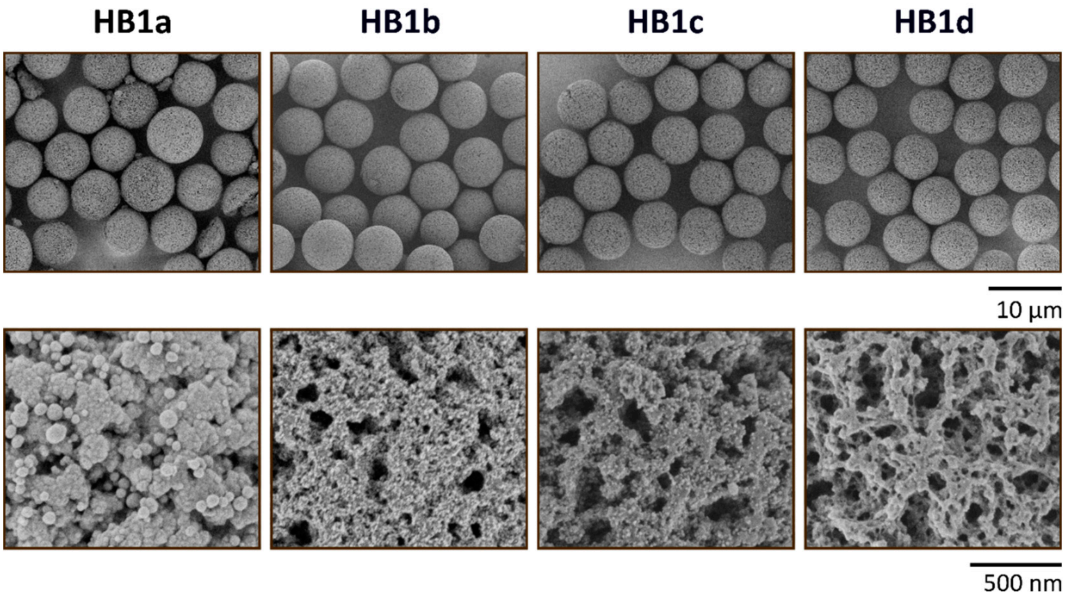

Figure S4. SEM images of hybrid beads **HB1a-d** with 2,000x magnification (top row) and 50,000x magnification (bottom row).

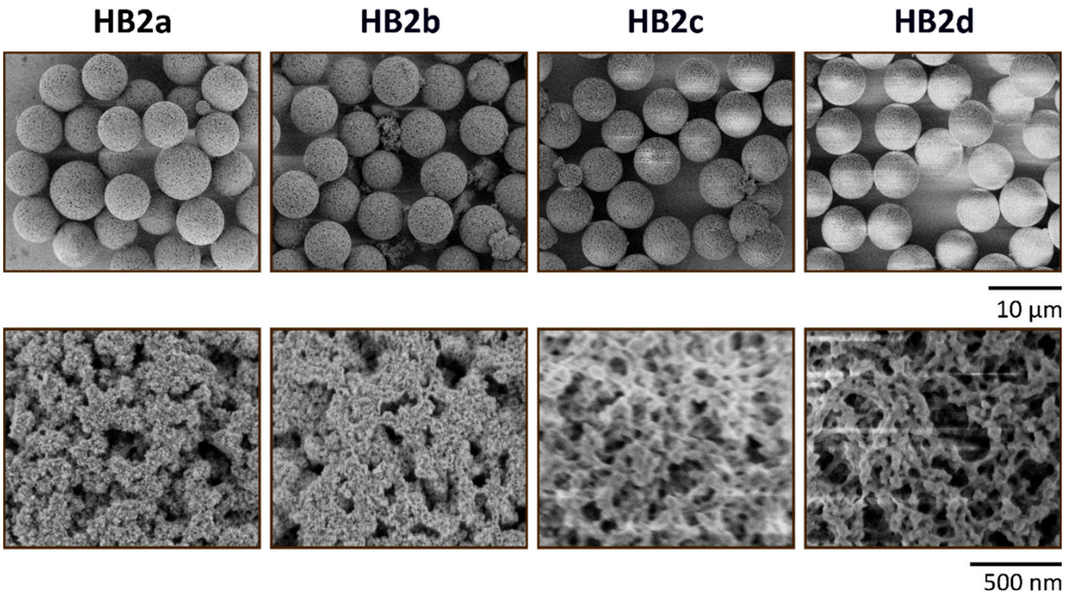

Figure S5. SEM images of hybrid beads **HB2a-d** with 2,000x magnification (top row) and 50,000x magnification (bottom row).

Table S1. Retention times of uracil and toluene after 1, 25, 50,75 and 100 runs in [min]

	1st run	25th run	50th run	75th run	100th run
t_r (Uracil)	3.31	3.31	3.33	3.31	3.28
t_r (Toluene)	4.03	4.03	4.05	4.01	3.98
